# Adapted psychotherapy for suicidal geriatric patients with depression

**DOI:** 10.1186/s12888-018-1775-y

**Published:** 2018-06-19

**Authors:** Jörn Conell, Ute Lewitzka

**Affiliations:** 1 AMEOS Klinika Neustadt, Lübeck und Eutin, Kliniken für Psychiatrie und Psychotherapie, 23730, Neustadt i.H, Germany; 2 Department of Psychiatry and Psychotherapy, University Hospital Carl Gustav Carus, Technische Universität Dresden, Fetscherstr. 74, 01307 Dresden, Germany

**Keywords:** Geriatric patients, Depression, Suicide, Psychotherapy

## Abstract

**Abstract:**

This debate article aims to evaluate whether current diagnostic and therapeutic options for suicidal geriatric patients with depression suffice, and which adapted strategies might be helpful. We hope to encourage clinicians to consider special approaches when treating the elderly.

**Background:**

Suicide in old age is a major public health problem, as the suicide rates are highest among those aged 60 years and older in most European countries. Although pharmacological treatment options are relatively easy for older patients to obtain, their access to standard psychotherapy is limited. The reasons for this are i) the widely shared attitude about the effectiveness of psychotherapy for older people and ii) the limited access to standard psychotherapy due to their immobility.

**Conclusion:**

New psychotherapeutic methods need to be developed. Psychotherapy at the patient’s home seems to be a new approach to accommodate that individual’s personal circumstances and make effective therapy possible.

## Background

An obvious problem in psychiatry and psychotherapy, and one inadequately researched despite its considerable relevance is the rise in suicide among the elderly. Although most individuals are quite content with their life as they age [1], suicide rates are very high among the elderly [2]. Although data from the US show that middle-aged adults have a higher suicide risk, older men remain the highest suicide risk group [3]. There must be biological, sociological, and psychological factors associated with advanced age of which we are unaware or to which we pay too little diagnostic and therapeutic attention [4]. Our health care services do not seem to be reaching the vulnerable group of depressed or suicidal senior citizens, or the affected elderly no longer feel the need to seek help in their situation. The latter factor probably contributes to this problem; negative stereotypes about old age permeate our perception and attitudes, as many of us prefer to avoid confronting the subject of aging. Other factors are that a) the elderly often live in social isolation, b) seldom take the initiative to discuss their emotional problems, and c) are seldom referred to specialists or psychotherapists by their general practitioners (GPs) [5].

The therapist’s task seems to be facilitated by the generally accepted assumption that age-related depression should be treated much like depression in younger adults – a fact mentioned in every textbook. We need to address specifics – taking depression among the elderly seriously could alleviate their emotional pain and even prevent suicide.

Hautzinger [6] describes particular characteristics of depression in old age, the most important being the experience of loss (of a partner or spouse, work, health). Especially those with little resilience who encounter stressful life events in old age tend to become depressed or commit suicide. Individuals who feel they have lost control over the changes that accompany aging are also particularly vulnerable.

Research is thus warranted into the reason why not all seniors who experience such life events become depressed or kill themselves. Other stress factors contributing to the development of depression in old age are physical infirmity, the loss of independence, needing constant care, and the loss of status. We believe that a multifactorial etiology plays an underlying role for this observation. One hypothesis is that some people’s ability to cope with difficulties during their life-span changes (e.g., influenced by depression); in some cases character traits may become reinforced or constricted, leading to less flexible coping skills.

With this article we aim to describe diagnostic and therapeutic strategies to reduce suicidality among the depressed elderly by relying on the latest evidence. Note that a detailed presentation of the complex issue of suicide in old age is not possible in this format, although we do address several key aspects thereof.

### Epidemiology of suicidality in the elderly

According to the WHO [7], about a million individuals worldwide take their own life per year, placing suicide among the 15 top causes of death globally across all age groups. We can assume that about 90% of those were suffering from a mental illness at the time of their suicide [8] (primarily from affective disorders). The American Foundation for Suicide Prevention (AFSP) maintains that about 28 times more individuals attempt suicide at some time [9].

When considering age groups, those 70 years of age or older of either gender have the highest suicide rate, an age distribution found nearly worldwide. One of the strongest risk factors for suicide in old age is having a psychiatric disorder [10]. The Berlin investigation of age demonstrated that 24% of those over age 70 present a mental illness and 33% admit to being under obvious emotional stress [11].

Psychological autopsy studies of individuals who died by suicide revealed that 71 to 97% were suffering from a psychiatric disorder, especially affective diseases [12]. Dementia is another apparent risk factor according to a longitudinal study by Erlangsen et al. [13].

The harboring of suicidal thoughts among senior citizens has not been well researched, and there is no statistical evidence. Burkhard et al. reported that about 5% of geriatric patients discussed getting help to commit suicide [14]. About two-thirds of the elderly who killed themselves had been seen by their GP the month before their death is remarkable (half of them were seen during the week beforehand) [15, 16].

### Insights about the causes of and therapy for Suicidality in advanced age

One of the reasons for older adults’ higher risk of committing suicide is their method of choice: they tend to use potentially more lethal methods. They are also more likely to be suffering from somatic diseases that lower the chance of surviving a suicide attempt. Furthermore, they tend to lead more socially isolated lives, thus first-aid measures often come much too late. Another factor is that older adults are less inclined to express their thoughts of suicide [17].

According to Van Ordern et al. [18], the elderly who attempt suicide employ harder and potentially more lethal methods when they feel they are no longer valued, a concept termed “thwarted belongingness”. Other reasons for suicides in the elderly are somatic problems, pain, the fear of becoming a burden, family conflicts, and the feeling that life no longer has a purpose.

The main cause of suicide in the elderly according to Lindner et al. [19] is physical illness, followed by interpersonal conflicts. Patients reported that they would be more apt to discuss suicide with their relatives than with professionals.

Conwell et al. [12] emphasize the major role of age-related depression as a pathogenetic factor in suicide among those of advanced age, calling for its early diagnosis and effective treatment.

Fiske et al. [20] investigated the relationship among control strategies, symptoms of depression and thinking about suicide in the elderly with health problems and found that those able to seek help thought less about it regardless of how depressed they were. They made similar observations in those able to persevere. They also discussed the motivational theory of lifespan development as particularly relevant to late life suicide [21].

Sachs-Ericsson et al. [22] investigated the causal relationship among suicidality, the severity of depression, and white matter lesions (WML) in the brain in the elderly by examining whether suicide attempts in a patient’s history correlated with more severe periods of major depression. Those who had attempted suicide had more WML on the left side. Previous suicide attempts were a predictor for a faster bilateral increase in the number of WML, which were likewise predictors of cognitive deficits. The authors surmise that cerebro-morphological changes result from the suicide attempt, which in turn may be a reason for their poor prognosis.

Richard-Devantoy [23] investigated the cognitive capacities of older patients who had attempted suicide with very dangerous means to other groups (e.g. with less dangerous methods). Those whose methods were more likely to be lethal exhibited poor cognitive control. Older patients at a high suicide risk seem incapable (because of having poor cognitive control) of handling real-life problems satisfactorily: “normal” problems are quickly perceived as a mass of catastrophic stressors.

Other studies investigated risk factors for suicidal ideation. Male gender, higher education, smoking, living alone, poor social support, no religious practice, financial strain, childhood physical abuse, history of suicide in the family, past or current mood/anxiety disorders, pain, poor self-perceived health and current use of antidepressants were found to be independently associated with suicidal ideation [24].

Hautzinger [6] defined goals for the psychotherapy of age-related depression: develop active, non-depressive behavior, reduce passive-depressive behaviors, correct those conditions favoring depression (such as isolation), correct behavior and resource deficits, develop behavior and attitudes that are situation-appropriate, eliminate and replace counterproductive attitudes and perceptions characterized by resignation or pessimism, stubbornness, inflexibility, and help to change expectations, learn to be both more accepting of uncomfortable facts and of the past. He and his team developed a psychotherapy that specifically targets depression in advanced age (DiA).

Gustafson [25] and his group showed that problem solving therapy (PST) was more effective than purely supportive treatment in reducing suicidal thoughts in older adults with depression and executive dysfunction. PST makes conceptual sense, as it has proven to be efficacious in alleviating depression, depression-associated activity restrictions, and mild cognitive deficits – all key suicide risk factors in older adults.

Lindner et al. [26] report on a psychodynamic psychotherapy by which very old adults are treated in their actual residential situation. This takes into account their immobility, which restricts their access to psychotherapy as it is now being practiced. Frequent topics addressed during psychotherapy sessions are illness, physical infirmity, the process of dying, and death itself.

Heisel et al. [27] revealed the efficacy of an interpersonal psychotherapy (IPT) adapted for older patients presenting a suicide risk.

### The psychotherapeutic care situation of older patients

In many western societies, special programs have been developed over the past few years to help inform the public about and improve the care of the elderly, ie, through education or workshops for specialists in geriatric medicine. This applies especially to the GP’s role and to geriatric care, eg, pain therapy. Nevertheless, we can assume that psychotherapy’s accessibility for older adults is insufficient. Very few validated, adapted programs exist. Kiosses et al. (2015) [28] developed PATH (Problem Adaptation Therapy), a home-delivered psychotherapy for older adults with major depression and cognitive impairment. They found that those patients participating in PATH revealed a significantly greater reduction in depression and disability.

There seem to be numerous reasons that those over the age of 60 fail to receive adequate psychotherapy.

For one, clinicians often note that especially older folks harbor reservations about mental illness and its treatment. The focus tends to be on physical infirmities and alleviating them. This is no doubt due to the stigmatization this age group associates with the diagnosis of mental illness. The second key reason has to do with this age group’s mobility restriction. While other service providers such as physiotherapists and ergotherapists are accustomed to treating patients in their homes, the strict official guidelines for psychotherapy mean that house visits are usually impossible in Germany.

Another reason worth considering is that many older adults maintain a negative attitude toward psychotherapy despite its growing acceptance and efficacy. Sigmund Freud ‘s view that older adults are by nature not receptive to psychotherapy (due to their no-longer-changeable personalities) found widespread acceptance for years among professional therapists, an attitude that is receding too slowly.

## Adapted psychotherapy

Suicide, suicide attempts, and suicidal thoughts are a serious and complex problem among our senior citizens.

We need various suicide-prevention strategies specifically targeting older patients suffering from depression. Their isolation is particularly relevant: innovative approaches are necessary that need not extend beyond the scope of health care providers, eg, by making “telehelp” available for socially isolated individuals [29].

A decisive step in our opinion is to actively approach older adults personally. This is especially important when a spouse has died and there are no family members to whom the surviving partner can turn. A key element in such a treatment protocol would be to ensure continuous visits by the psychosocial services; somatic or psychiatric therapists need not play the main role. If risk factors for suicide or depression such as thwarted belongingness, conflicts, feeling there is no reason to go on living – if any of these factors comes to light through such low-key contact, the next step would be to involve the patient’s GP.

GPs play an essential role in this age group’s lives, and they need to be actively recruited and schooled in the clinically often multifactorial symptoms of age-related depression and its treatment. We need to encourage their cooperation with psychiatric and psychotherapeutic professionals. They should also establish contact with the family, who are often the first to hear about any suicidal thoughts.

Established programs such as DiA, PST, and IPT should play an important role in the psychotherapy of older adults. Group therapy formats should be researched and promoted where applicable. There is no evidence that specific group therapies – when accommodating needs of the elderly –cannot be similarly effective as they are in younger patient groups. Such groups would certainly help older adults make new acquaintances and contacts, who would continue to play a helpful role once the therapy has concluded. A more active social life can help restore self-confidence and make life more meaningful.

Psychotherapy measures must take the changes in cognitive capacity associated with old age into account: it may take longer to achieve certain goals. As Richard-Devantoy [23] demonstrated so impressively, some set goals may eventually prove to be thoroughly unrealistic in light of the limited cognitive abilities of some older depressed and suicidal individuals. Such patients may end up in an even more desperate situation if they feel the therapy is being forced them or they find it overwhelming, as they are not cognitively equipped to develop effective alternative strategies. Rather than insist on a strict 50-min session, the therapist should be sensitive to the patient’s state-of-mind at each encounter and judge whether offering shorter and thus more frequent meetings would be more productive.

A significant number of the elderly are excluded from psychotherapy simply because of their restricted mobility; few psychotherapy practices are, for instance, wheelchair-accessible. The guidelines for psychotherapy in Germany need to be liberalized too. This applies to where the therapy takes place (ie, the patient’s home), its duration, frequency, and the total number of sessions. It remains to be seen how effective the changes to the psychotherapy guidelines made on April 1st 2017 are [30]. They have introduced a psychotherapy consultation to improve the access to psychotherapy by emotionally ill older citizens presenting acute symptoms.

It would make sense to include cranial magnetic resonance diagnostics; older patients with proven vascular brain damage who have already attempted suicide are at a high risk of killing themselves.

Age-related depression is a treatable condition that can range from being fed up with life to harboring concrete thoughts of committing suicide. These patients need urgent attention – both psychiatric-psychopharmacological and psychotherapeutic care.

## Conclusion

Reducing age-related suicide is a medical and societal challenge. Hidden or inadequately treated depression is often the reason behind such loss of life. Granted, the access to this patient population is problematic. The status of psychotherapy for older depressed adults must rise. This is possible by adapting therapies, ie, by designing low-key screening programs run by the psychosocial services, or by having GPs refer their vulnerable patients promptly to psychotherapists for treatment (at home if necessary) while considering the patient’s actual condition. Health factors that need to be considered are the patient’s cognitive health and any vascular brain damage. Age-suitable and well-tested therapy protocols need to be applied, and strict psychotherapy guidelines need to be relaxed.

## Abbreviations

AFSP: American Foundation for Suicide Prevention
DiA: Depression in advanced Age
GP: General Practitioner
IPT: Interpersonal Psychotherapy
PST: Problem Solving Therapy
WHO: World Health Organization
WML: White Matter Lesions
